# Enhancing the Mechanical Properties of 3D-Printed Waterborne Polyurethane-Urea and Cellulose Nanocrystal Scaffolds through Crosslinking

**DOI:** 10.3390/polym14224999

**Published:** 2022-11-18

**Authors:** Julen Vadillo, Izaskun Larraza, Tamara Calvo-Correas, Loli Martin, Christophe Derail, Arantxa Eceiza

**Affiliations:** 1 “Materials + Technologies” Research Group (GMT), Department of Chemical and Environmental Engineering, Faculty of Engineering of Gipuzkoa, University of Basque Country, Plz. Europa 1, 20018 Donostia-San Sebastian, Spain; 2 Universite de Pau et des Pays de l’Adour, E2S UPPA, CNRS, IPREM, 64000 Pau, France; 3 CIDETEC, Basque Research and Technology Alliance (BRTA), Po. Miramón 196, 20014 Donostia-San Sebastián, Spain; 4 Macrobehavior-Mesostructure-Nanotechnology, General Research Service (SGIker), Polytechnic School, University of the Basque Country, Plaza Europa 1, 20018 Donostia-San Sebastián, Spain

**Keywords:** waterborne polyurethane-urea, 3D printing, mechanical properties, crosslinking, scaffolds, cellulose nanocrystals

## Abstract

In this work, shape-customized scaffolds based on waterborne polyurethane-urea (WBPUU) were prepared via the combination of direct ink writing 3D-printing and freeze-drying techniques. To improve the printing performance of the ink and guarantee a good shape fidelity of the scaffold, cellulose nanocrystals (CNC) were added during the synthesis of the WBPUU and some of the printed constructs were immersed in CaCl_2_ prior to the freeze-drying process to promote ionic crosslinking between calcium ions and the polyurethane. The results showed that apart from allowing the ink to be successfully printed, obtaining scaffolds with good shape fidelity, the addition of the CNC resulted in a greater homogeneity of the porous structure as well as an increase of the swelling capacity of the scaffolds. Additionally, the CNC has a reinforcement effect in the printed systems, presenting a higher compression modulus as the CNC content increases. In the case of samples crosslinked by calcium ions, a rigid shell was observed by scanning electron microscopy, which resulted in stiffer scaffolds that presented a lower water absorption capacity as well as an enhancement of the thermal stability. These results showed the potential of this type of post-printing process to tune the mechanical properties of the scaffold, thus widening the potential of this type of material.

## 1. Introduction

Direct ink writing 3D printing (DIW), also known as extrusion-based 3D printing, is a novel layer-by-layer assembly technique that can be used to build structures designed by a computer [1,2]. This technology, which consists of a piston or a screw that extrudes the ink through a nozzle [3,4], has gained increasing attention in the last years, due to the possibility of printing customized designs overcoming drawbacks of classic 3D printing, such as the use of organic compounds or temperature requirements [5]. However, in the DIW printing technique, the viscosity rheological performance of the ink needs to be carefully controlled [6,7]. The ink design requires to consider the viscosity, yield point under shear, and viscoelastic properties (elastic and loss moduli) [8]. Concretely, inks must present a shear thinning behavior to be easily extruded [9,10], but also a well-defined yield point and a high storage modulus, to allow the shape retention after deposition [11,12]. To achieve this complex behavior, one can detect different approaches in the literature. One of them consists of the incorporation of additives such as cellulose nanocrystals (CNC), for example, to pilot rheology [13,14,15], and/or controlling the printing temperature [7], but also to enhance the shape fidelity of the printed piece through chemical crosslinking after the printing process.

Regarding the scaffold preparation, the freeze-drying technique is extensively used to prepare porous materials from stable polymer emulsions in aqueous medium. However, as reported in the literature, a non-aqueous co-solvent can also be used, presenting benefits such as decreased drying time or increased stability of the dry product, but it also has important disadvantages such as toxicity or a high degree of flammability risk [16]. The principal advantage of freeze-drying is the formation of porous materials, avoiding the use of organic solvents [17], and the elimination of time-consuming drying and leaching processes of porogen components. However, the instability of the emulsion, which requires the addition of suitable surfactants, represents the principal problem in the preparation of scaffolds by this method [18]. In this context, the use of waterborne polyurethanes (WBPU) and waterborne polyurethane-urea (WBPUU) represents an alternative to overcome this drawback, as was reported in [19], since the inclusion of an emulsifier in the polymer backbone allows the formation of stable emulsions [20], allowing the successful preparation of the scaffold through freeze-drying. Du et al. successfully prepared scaffolds parting from a WBPU containing PLGA, which presented a controllable degradation rate [21], whereas Lin et al. obtained WBPU scaffolds via freeze-drying with an ordered architecture, presenting great potential in tissue engineering for biological anisotropic tissue regeneration [22].

One of the principal drawbacks of most of these types of inks is the poor mechanical performance that the final material exhibits [23], where, according to the literature, the incorporation of reinforcements is the most followed strategy for its enhancement [24,25,26]. In this context, many recent works have explored the use of fillers in order to improve the mechanical properties of printable materials in different additive manufacturing techniques, such as calcium silicate hydrate filler in acrylated epoxidized soybean oil (AESO), commercial photocurable resin for digital light processing (DLP) [27], or hydroxyapatite and titanium oxide in polylactic acid for fused deposition modeling (FDM) [28], among others. For DIW, for example, Li et al. obtained a printable ink with better printability and mechanical properties by adding SiO_2_ particles to a polyamide acid-based ink [29]. In these works, an enhancement of the mechanical properties was observed in general, however, as observed by Hada et al., this mechanical modification is influenced by the filler content, as expected [30]. However, as observed in the literature, the use of fillers in additive manufacturing applications can present problems, such as aggregation of particles, which can affect the printing resolution [31], leading to the requirement of additional procedures to properly disperse the filler, such as sonication [32]. In contrast, the use of hydrophilic CNCs, which disperse in aqueous media, favors integration in the aqueous dispersion, such as WBPU [33]. Moreover, when integrated in situ, at an early stage of synthesis, the aforementioned dispersion problems can be avoided [34].

Additionally, as shown in many works, functionalization of the fillers was required to improve the adhesion with the matrix and thus improve the reinforcement effect of the filler [35,36,37]. In the case of DIW, Jian et al. obtained a modification of the mechanical properties by adding surface-modified flax fiber to a silicon elastomer [38]. Regarding nanocellulose, Larraza et al. obtained WBPUU-based inks suitable for DIW by adding different carboxylated cellulose nanofibers both in situ and ex situ. The rheological behavior and final properties were fine-tuned by varying the type of nanocellulose, the content, and the addition method. The reinforcing effect of cellulose nanofibers increased as the degree of carboxylation increased, due to the higher number of interactions between WBPUU and carboxylated nanofibers [34]. Apart from that, other authors explored chemical processes after the printing to improve the mechanical properties of the resulting material. Chemical crosslinking with calcium chloride is a common gelation method for alginate-based inks [39], resulting in obtaining calcium alginate, which has presented many uses in wound-dressing applications [40,41]. However, other works remark on the possibility of using this technique to promote chemical crosslinking in other polymers, such as previously functionalized polyethylenglycol (PEG) [42] or a branched polymer synthesized from maltodextrin and octenyl succinic anhydride (OSA) [43], among others. Specifically, and focusing on the WBPU and WBPUU, Huang et al. studied the influence of the Ca^2+^ crosslinking in waterborne polyurethanes [44]. This chemical crosslinking was promoted between the calcium ions and carboxylate groups of WBPU, as was also observed previously by Lin et al. [45]. The resulting material presented higher density, storage modulus, thermal stability, and tensile strength than the not crosslinked one.

Hence, in this work, different inks composed of waterborne polyurethane-urea and cellulose nanocrystal nanocomposites were printed by DIW 3D printing and subsequently freeze-dried to prepare shape-customized scaffolds. Some of the printed pieces were immersed in CaCl_2_ before the formation of the porous scaffold through freeze-drying to establish a chemical bonding between the WBPUU and the Ca^2+^. The ionic crosslinking of WBPUU through calcium ions should result in a modification of the morphology of the systems, leading to more rigid pieces, as was observed by other authors [44]. The prepared scaffolds have been analyzed physicochemically, morphologically, and mechanically to study the influence of both CNC and of the crosslinking.

## 2. Materials and Methods

### 2.1. Materials

WBPUU inks were synthesized with a soft segment (SS) composed by two di-functional polyols, polycaprolactone (PCL) (CAS: 24980-41-4) and poly (ethylene glycol) (PEG) (CAS: 25322-68-3) (Mn = 2000 and 1000 g mol^−1^, respectively), both provided by Sigma-Aldrich. On the contrary, 2,2-bis(hydroxymethyl) propionic acid (DMPA, 98% purity, CAS: 4767-03-7) and ethylene diamine (EDA, 99% purity, CAS: 107-15-3), which were used as an internal emulsifier and a chain extender, respectively, also provided by Sigma-Aldrich, and isophorone diisocyanate (IPDI, 99.5% purity, CAS: 4098-71-9), supplied from Covestro, were used as components of the hard segment (HS). Dibutyltin dilaurate (DBTDL, CAS: 77-58-7), provided by Sigma-Aldrich, was used as a catalyst. The polyols and the DMPA were dried under a vacuum at 60 °C for 4 h prior to their use. Butanone (supplied by Panreac, CAS: 78-93-3) was used to adjust the viscosity of the prepolymer in an intermediate state as well as to transfer the neutralized DMPA into the reaction medium. The neutralization of the carboxylic groups of DMPA has been carried out by using triethylamine (TEA, 99.5% purity, CAS: 121-44-8), provided by Fluka. Cellulose nanocrystals (CNC) provided by the University of Maine (2018-FPL-CNC-117) were used and CaCl_2_ solution (0.1 M, CAS: 10043-52-4) was provided by Sigma-Aldrich.

### 2.2. Preparation of WBPUU/CNC Nanocomposite-Based Inks

The synthesis of WBPUU/CNC nanocomposites has been carried out following the procedure described in a previous work [46]. The procedure consists of the synthesis of the WBPUU in a two-step process, where the nano-entities are incorporated during the synthesis of the matrix itself. In a first step, the PCL, PEG, and IPDI were mixed at 80 °C during 3 h under the presence of 0.1 wt.% of DBDTL. Then, the system was cooled until 60 °C and the DMPA, previously neutralized with TEA and dissolved in butanone, was added, leaving to react for 1 h. Thereafter, the formed prepolymer was cooled until room temperature, where the second part of the synthesis was carried out. In this second step, the CNCs were added, and dispersed in deionized water drop-by-drop under vigorous stirring. In this way, the addition of the nano-entities and the particle formation of the WBPUU are produced at the same time. For the matrix not containing CNC, only deionized water was added at this step. Once the CNC were added, the system was heated up to 35 °C and EDA was added, previously dissolved in deionized water, being incorporated drop-by-drop under low stirring. Finally, the WBPUU reacted during 2 h, and the butanone was removed from the resulting dispersion by vacuum distillation (2 h at 60 °C).

The WBPUUs were synthesized in a ratio of 0.8/0.2/3.5/1/1.5 (PCL/PEG/IPDI/DMPA/EDA), with a solid content of 29 wt.%. According to previous works, inks with this solid content do not show any capacity to maintain the given shape after being printed [47], however, they allow the incorporation of CNC in situ, resulting in inks with good printability by DIW 3D printing, obtaining a good shape fidelity in the printed pieces [46]. Regarding the CNC incorporation, WBPUU/CNC nanocomposites were prepared containing 0.25 and 0.5 wt.% of nano-entities.

### 2.3. DIW 3D Printing of the Inks and Scaffold Preparation by Freeze-Drying

WBPUU and WBPUU/CNC inks were printed by DIW 3D printing as cylinders of 12 mm in diameter and 5 mm in height. The printing process was carried out using a Voladora 3D-printing machine, provided by Tumaker (Spain), which has been adapted to allow the printing of inks by DIW 3D printing. As a previous step of the printing process, the different inks were loaded into syringes and were centrifuged (3 min at 3000 rpm) to remove the existing bubbles and avoid the posterior apparition of defects in the printed parts. Regarding the printing conditions, the samples were printed using a needle of 0.8 mm in diameter at a printing speed of 6 mm s^−1^.

**Figure 1:**
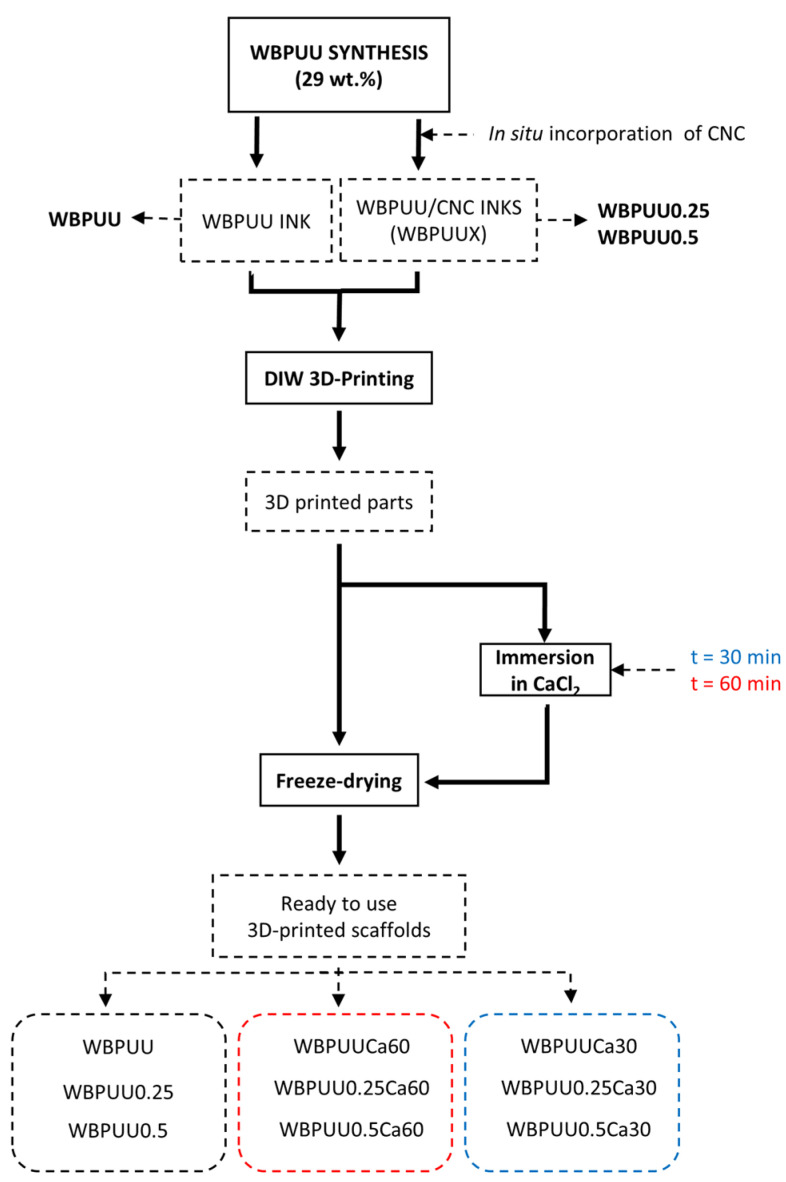
Flow chart of the experimental procedure for the preparation of 3D-printed scaffolds.

The stabilization of printed parts’ shape was performed by freeze-drying. Prior to the freeze-drying process, some of the samples were immersed in a CaCl_2_ solution of 0.1 M for 30 min or 1 h to introduce crosslink in the printed piece. After this, the samples were washed with deionized water to remove the residual CaCl_2_. Finally, porous scaffolds were obtained by freeze-drying. Initially, the pieces were frozen for 24 h at −40 °C and subsequently freeze-dried (−80 °C and 0.1 mbar) for 3 days. In Figure 1, a scheme of the experimental procedure is displayed.

The designation, composition, and preparation process of all prepared scaffolds are listed in Table 1.

**Figure 2:**
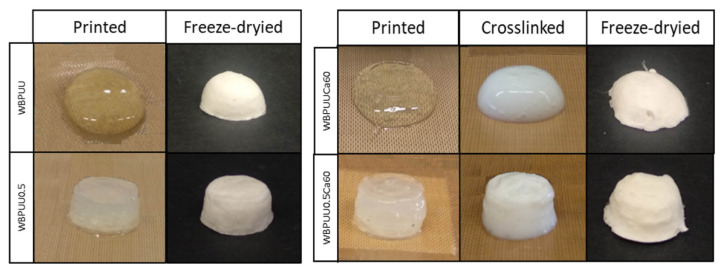
Images of the prepared scaffolds at different stages for WBPUU and WBPUU0.5 (**left**) and WBPUUCa60 and WBPUU0.5Ca60 (**right**).

As a result of the freeze-drying process, scaffolds with a cylindrical form were obtained. In Figure 2, digital images of the scaffolds are shown before and after the freeze-drying process for systems with/without CNC and with/without immersion in CaCl_2_ prior to scaffold formation. As can be observed in Figure 2, systems containing CNC showed a good reproducibility of the 3D cylindrical design after printing, which corroborated the results obtained in a previous work where the in situ incorporation of CNC improved the shape fidelity of the printed piece [46]. However, after the freeze-drying process, the form obtained from WBPUU seems to lose definition even more, with a rounding of the edges, as shown in Figure 2. Additionally, in samples which were previously immersed in CaCl_2_ before freeze-drying, a loss of definition was also observed compared to those that were not immersed in CaCl_2_, due to the ionic bond formation during the crosslinking process and subsequent contraction. This loss of definition is again much higher in the samples without CNC addition.

The immersion of the printed piece in CaCl_2_ resulted in the apparition of a white-colored shell in the piece, which presented high rigidity and was able to support up to 50 g even before the freeze-drying process. This rigid shell is formed due to a chemical crosslink produced between the COO^−^ groups of the WBPUU and the Ca^2+^ of the calcium chloride, as proposed by Huang et al. [44].

### 2.4. Characterization

The rheological measurements of the inks immersed and not immersed in CaCl_2_ were carried out using a dynamic rheometer (R101, Antoon Paar). Viscosity as a function of time was obtained by rotational rheology at 22.5 °C using a plate–plate geometry of 25 mm, with a shear rate fixed at 1 s^−1^ during 180 s. For the determination of storage and loss moduli (G′ and G″, respectively), spectromechanical analysis was performed at 22.5 °C using a plate–plate geometry of 25 mm, with a fixed strain and frequency of 1% and 1 Hz, respectively, during 180 s.

The characteristic functional groups of the scaffolds prepared from WBPUU and WBPUU/CNC (both immersed and not immersed in CaCl_2_) were analyzed by Fourier transform infrared spectroscopy (FTIR) using a Nicolet Nexus spectrometer provided with a MKII Golden Gate accessory (Specac), with a diamond crystal at a nominal incidence angle of 45° and a ZnSe lens. Spectra were recorded in attenuated reflection (ATR) mode between 4000 and 650 cm^−1^, averaging 64 scans with a resolution of 8 cm^−1^.

The thermal stability of the prepared scaffolds was determined by thermogravimetric analysis (TGA) using Mettler Toledo equipment (TGA/STDA 851). Between 5 and 10 mg of samples were introduced in ceramic pans. The samples were heated from 25 to 800 °C in N_2_ atmosphere at a scanning rate of 10 °C min^−1^. From the obtained degradation curves and their derivatives, the initial degradation temperature (T_0_), as the loss of 5% of the initial weight, and the maximum degradation temperature (T_d_), as the minimum of the degradation peak in the derivative of the thermogravimetric curve, were determined.

To study the morphology of the prepared scaffolds, scanning electron microscopy (SEM) measurements were performed by a Field-Emission Gun Scanning Electron Microscopy (FEG-SEM), Hitachi S-4800N, at a voltage of 5 kV. The images were obtained using a combination of a TTL and an Everhart–Thornley (ET) detector. Prior to the test, and to analyze the cross-section of the prepared scaffolds, the samples were cryo-fractured in liquid nitrogen to avoid the deformation of the scaffolds during the cutting process and sputter-coated with a thin layer of gold (~10 nm^−9^ in an Emitech K550X ion-sputter).

In addition, to determine the elemental composition of the different areas, energy-dispersive X-ray spectroscopy (EDS) was carried out by means of an ULTRAplus scanning microscope (SEM) from Zeiss Company equipped with a Gemini column and with an energy-dispersive X-ray spectrometer from the OXFORD INCA Synergy microanalysis system.

The compression properties of the WBPUU- and WBPUU/CNC-based scaffolds were determined by testing the prepared cylinders at room temperature with an Instron 5967 universal testing machine. The compression force was applied in the layer-by-layer printing direction and the samples were compressed at a fixed length of 4 mm at a crosshead speed of 10 mm min^−1^. The average value of the elastic compression modulus was calculated as the slope of the stress–strain curve at low deformations. The compressive strength was taken as the stress reached at a strain of 40% for all samples and the densification strain was taken as the strain at the intersection point between the stress plateau and a line extrapolated from the densification line. All these values were averaged for three specimens. The specific modulus and specific stress were determined as the ratio between the Young’s modulus and the density, and the stress at a strain of 40% and the density, respectively.

The density of the scaffolds was obtained as the ratio between the weight and volume of the printed cylindrical specimen and values were averaged for five specimens.

The evolution of the water absorption capacity (WAC) of the scaffolds was carried out by weight difference measurement to analyze the capacity of the systems to absorb water. For this study, specimens of around of 10–15 mg were immersed in 5 mL of deionized water at 25 °C. Samples were weighted at different times until stabilization. To study the influence of the pH on the absorption capacity of the scaffolds, different pH values of 1, 7, and 12 were used. For this purpose, hydrochloric acid (HCl 0.1 M) and sodium hydroxide (NaOH 0.1 M) were used to adjust the pH to 1 and 12, respectively. The water absorption content was determined from the weight increase by means of Equation (1):

(1) $$WA\;\left(\%\right)=\frac{W_{t}-W_{0}}{W_{0}}\times 100$$

where *W_t_* is the weight at time *t*, whereas *W*_0_ is the initial weight of the sample. Three measurements were averaged for each sample.

## 3. Results and Discussion

### 3.1. Influence of the CaCl_2_ Immersion of the WBPUU/CNC Inks

**Figure 3:**
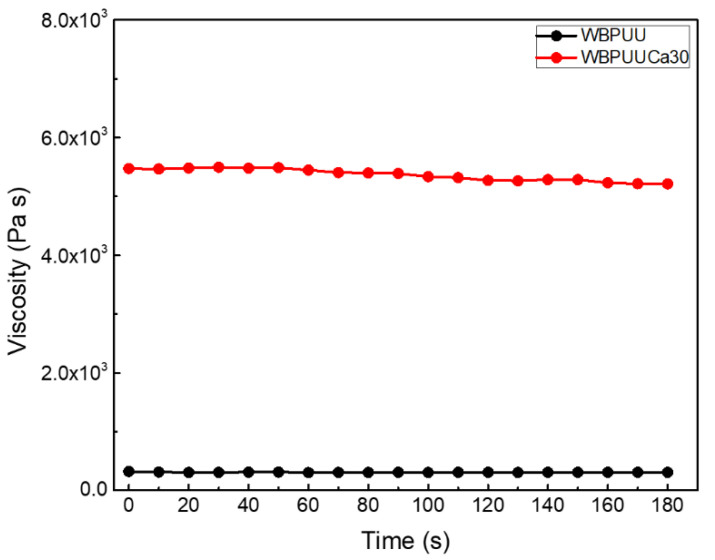
Viscosity as a function of time at a shear rate of 1 s^−1^ (T = 22.5 °C) for WBPUU before and after the immersion in CaCl_2_.

The consistency of the crosslinking process of the inks after the immersion into CaCl_2_ solution was tested by rheology analysis. For that, flow tests were carried out for a WBPUU ink prior to and after the immersion to study the influence of the aforementioned crosslinking procedure into the viscosity of the inks. The results, which are displayed in Figure 3, showed a clear increase of the viscosity of the crosslinked system, presenting a constant viscosity value of around 300 Pa·s for the unreacted WBPUU ink, in contrast with the value of around 5200 Pa·s of the ink immersed in CaCl_2_. The crosslinking process produced between the Ca^2+^ and the COO^−^ groups of the WBPUU particles resulted in an increase of the viscosity due to the chemically crosslinked network.

**Figure 4:**
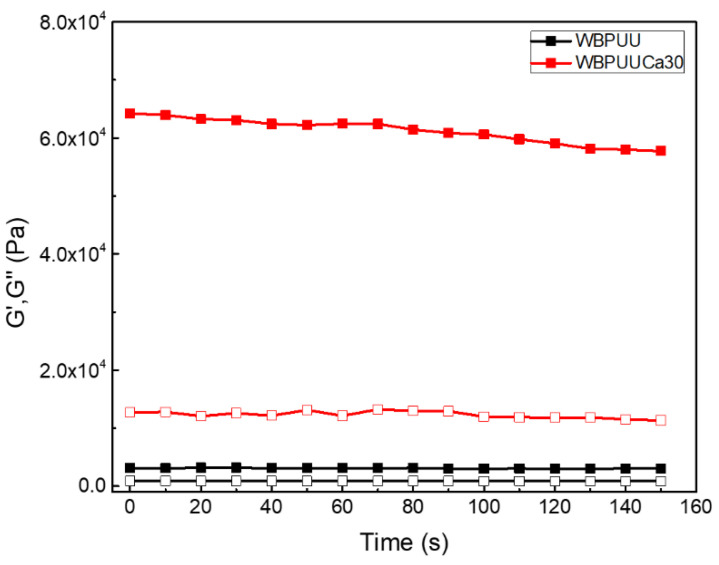
Storage (■) and loss (□) moduli as a function of time at a frequency of 1 Hz and a strain of 1% (T = 22.5 °C) for WBPUU before and after the immersion in CaCl_2_.

The storage and loss moduli of both not crosslinked and crosslinked WBPUUs as a function of time are reported in Figure 4. The crosslinking with Ca^2+^ resulted in an increase of both storage and loss moduli, compared with the not crosslinked system. The systems studied after the crosslinking process presented an increase of around 1900% and 1400% of the storage and loss moduli at 1 Hz, respectively. According to the literature, a high storage modulus is required for a good shape fidelity, requiring values above 10^3^ for optimum shape fidelity [6]. Thus, via this process, a higher storage modulus can be achieved even for inks presenting low G′. Additionally, the tan, δ, of the systems tested after the immersion in CaCl_2_ solution presented lower values compared with the ones observed for the not crosslinked WBPUU (0.2 for the WBPUUCa30 vs. 0.3 for WBPUU). This evolution towards lower values of tan, δ, illustrates the transition between an initially viscous behavior of the not crosslinked WBPUU and the more elastic behavior observed in the WBPUU after the immersion in CaCl_2_.

### 3.2. Characterization of the WBPU/CNC Prepared Scaffolds

**Figure 5:**
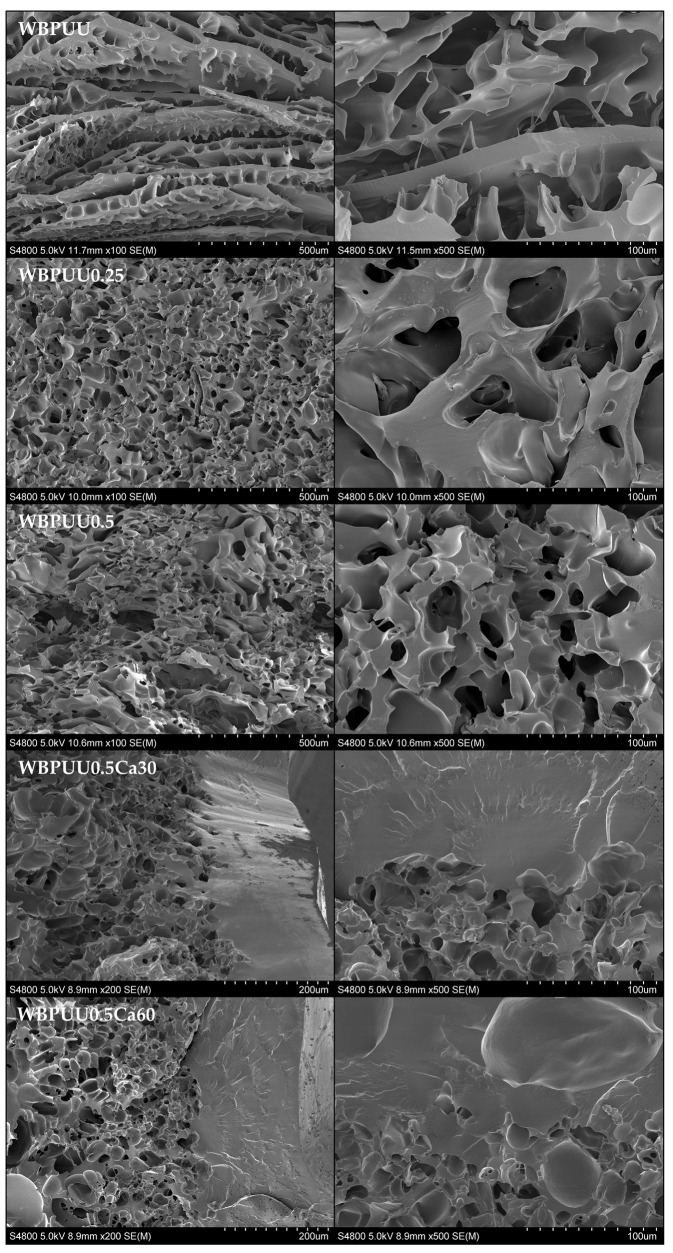
SEM images of the different WBPUU/CNC and WBPUU/CNC scaffolds immersed in CaCl_2_.

To study the morphology of the prepared scaffolds, scanning electron microscopy was used to obtain images of the inner structuration of the printed scaffolds. The images taken in the transversal section of the scaffolds are displayed in Figure 5. Analyzing the obtained results, a heterogeneous morphology can be observed for the WBPUU-based scaffold. The liquid-like behavior of this ink is not able to support a multilayered 3D construct, resulting in the collapse of the structure, leading to obtaining the aforementioned heterogeneous morphology. Regarding the systems containing CNC, both WBPUU0.25 and WBPUU0.5 presented a homogeneous morphology, showing a higher structuration of the inner structure of the scaffold. In this case, the printed inks are able to support the weight of the upper layers, allowing to obtain multilayered ordered structures, which resulted in a homogenous morphology after the freeze-drying. Comparing the porosity of the scaffolds presenting different CNC content, the pore size seemed to decrease as the CNC content increased, resulting in the system presenting a higher specific surface. Similar results were found in the literature, attributed to the formation of physical crosslinking with the matrix [48].

**Figure 6:**
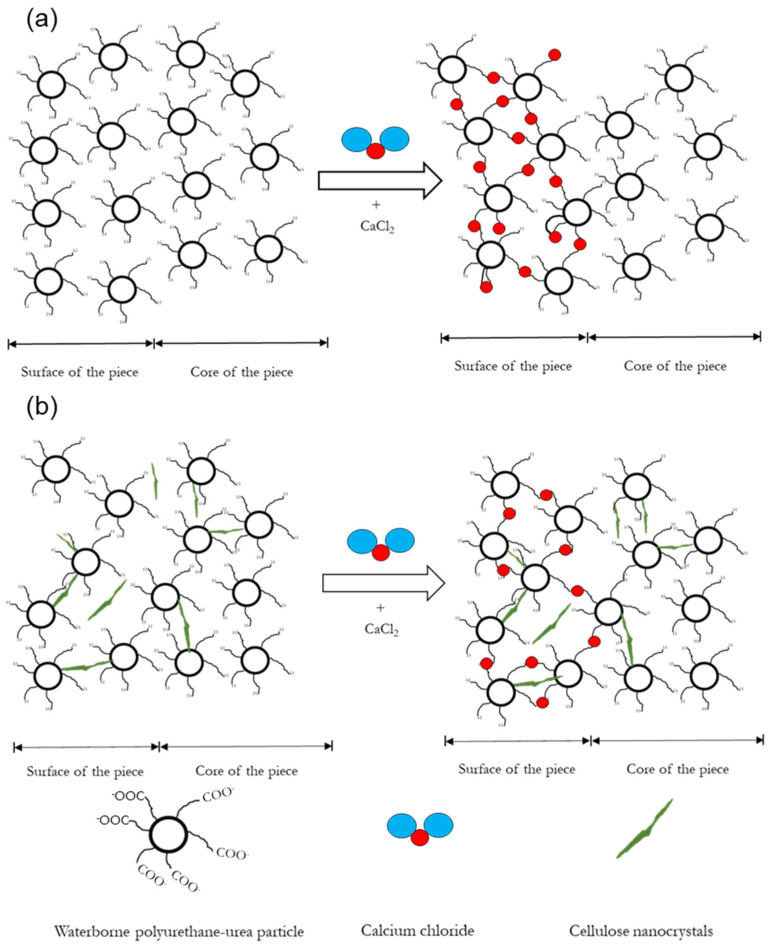
Proposition of a scheme of the crosslinking process of (**a**) WBPUU pieces immersed in CaCl_2_ and (**b**) WBPUU/CNC pieces immersed in CaCl_2_.

According to the images, the immersion of the printed scaffolds in CaCl_2_ prior to the freeze-drying process resulted in the formation of a solid shell of around 200 μm at the surface of the piece as a consequence of the chemical crosslinking by Ca^2+^. In Figure 6, a scheme of the crosslinking developed at the surface of the piece is proposed, in both WBPUU and WBPUU/CNC systems, as a result of the immersion in CaCl_2_. As shown, the carboxylate ions of the WBPUU at the surface of the printed piece react with the Ca^2+^ ions, in contrast to the core of the piece.

As a result of this crosslinking, the porosity observed for the non-crosslinked systems disappeared completely at the surface. The crosslinked WBPUU presented more interactions between particles, thus reducing the space between them and, therefore, the size of the ice crystals that will form the pores during the freeze-drying process. Finally, as a result of this formed shell, the previously observed homogeneous morphology in systems containing CNC seemed to disappear, observing an increase of the pore size. In this case, the aforementioned shell may have hampered the freeze-drying process, and thus scaffolds with a more heterogeneous morphology were obtained.

Additionally, to confirm the presence of Ca^2+^ ions and the aforementioned crosslinking in the shell of the scaffold, EDS analysis was performed on both core and shell zones in a WBPUU0.5Ca60 system. The results, displayed in Table 2, showed the presence of the Ca in the composition of the shell, which was not observed in the core, confirming the presence of Ca^2+^ on the surface of the printed scaffold immersed in CaCl_2_, thus forming a rigid, non-porous shell. Additionally, a residual amount of Cl was also observed in the shell, which was not observed in the core.

**Figure 7:**
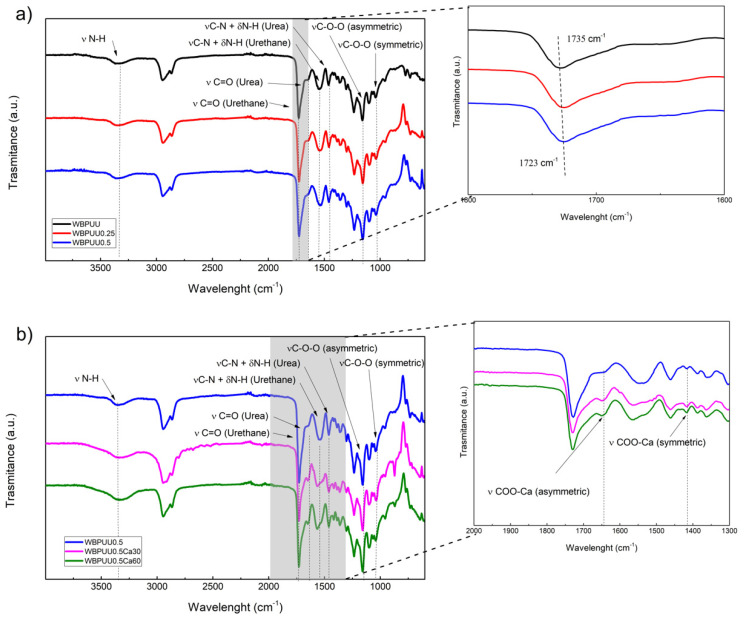
Spectra of the prepared scaffolds: (**a**) WBPUU scaffolds with different CNC content, and enlargement of the 2000–1400 cm^−1^ interval of (**a**) (inset), and (**b**) WBPUU/CNC scaffolds with different immersion times in CaCl_2_, and enlargement of the 2000–1300 cm^−1^ interval of (**b**) (inset).

The chemical structure of the prepared scaffolds was studied by FTIR to analyze the interactions between CNC and WBPUU functional groups and the effect of the crosslinking with Ca^2+^. The obtained spectra of the different inks are displayed in Figure 7.

All scaffolds showed characteristic bands of aliphatic polyester-polyether waterborne polyurethane-urea (Figure 7a), which are listed in Table 3. Analyzing the obtained spectra for systems with increasing CNC content, a slight shift in the carbonyl band can be observed towards lower wavelengths (inset of Figure 7a), from 1735 to 1723 cm^−1^, indicating an interaction occurring between the CNC and the WBPUU matrix, as also observed previously [46].

Concerning the effect of the crosslinking through calcium, the spectra of the samples immersed in CaCl_2_, which are shown in Figure 7b, exhibited a similar spectrum compared with the not crosslinked ones, however, slight differences can be observed in crosslinked systems in the band attributed to the stretching vibration of carboxylate. According to the literature, the carboxylate salt ion should present a signal around 1610 cm^−1^. In the case of calcium, the band attributed to the asymmetric stretching vibrations of the carboxylated salt ion is located in the 1600–1700 cm^−1^ range, as has been reported for calcium alginate [59,60,61]. In this case, samples immersed in calcium chloride showed a clear band at 1648 cm^−1^, as can be observed in the magnification of the 2000–1300 cm^−1^ band (inset of Figure 7b), and can be assigned to the aforementioned asymmetric COO-Ca bond. Conversely, the peak situated around 1420 cm^−1^ can be related to the symmetric COO-Ca bonding, as was described in the literature [59,60,61], which situated this signal in the range between 1440 and 1410 cm^−1^.

**Figure 8:**
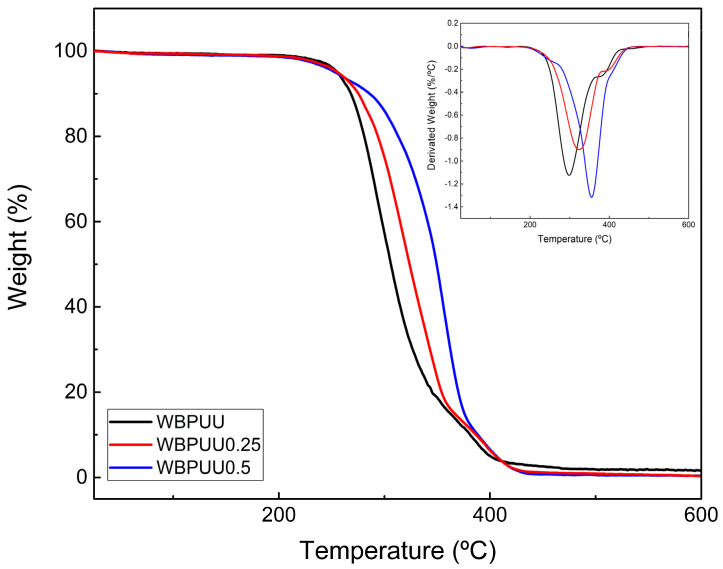

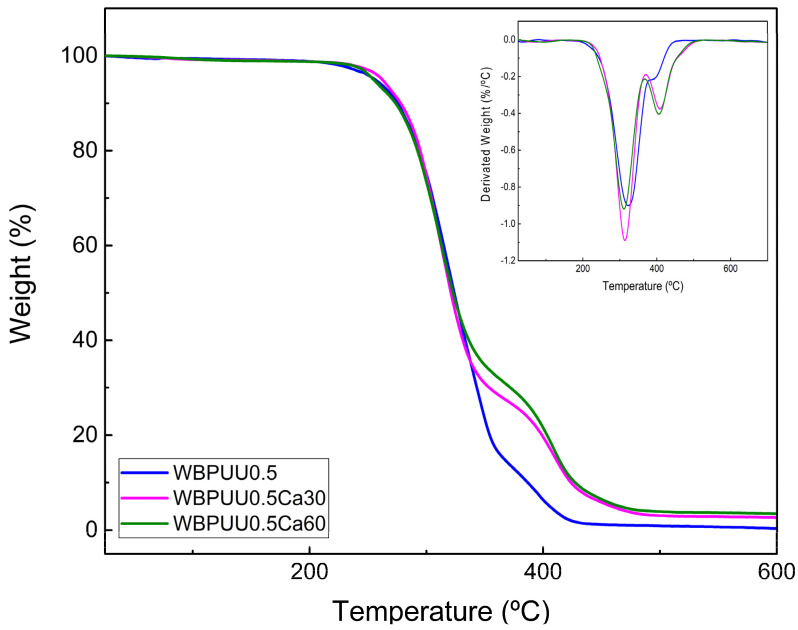
Variation of the weight of systems containing different CNC content (**top**) and of systems with different immersion times in CaCl_2_ (**bottom**). Weight loss derivative is displayed in the inset of each figure.

Moreover, the thermal stability of the different WBPUU and WBPUU/CNC scaffolds has been tested by TGA. The obtained degradation curves, as well as their derivative curves, are shown in Figure 8. The results showed a two-step degradation process for all WBPUU/CNC systems, as was also previously observed in films prepared with the same matrix [62]. Regarding the influence of the CNC, their addition resulted in an increase of the thermal stability due to the stabilization of urethane groups by interactions between WBPUU and CNC. Moreover, an enhancement of the thermal resistance with the CNC content was observed.

On the contrary, the immersion in CaCl_2_ did not seem to improve the thermal stability, presenting just a slight increase with the immersion time, in accordance with the results obtained by Huang et al. for a WBPU-Ca crosslinked system [44]. In addition to this, in the DTG curves of the samples immersed in CaCl_2_, a new peak can be appreciated around 400 °C (inset of Figure 8, bottom). According to the literature, the addition of Ca^2+^ seems to increase the thermal stability when it is crosslinked with carboxyl salt ion groups, as was observed by different authors for the calcium alginate [60,63,64]. In this case, the crosslinked shell of the systems immersed in CaCl_2_ presented a higher thermal stability compared with the unreacted core, which can be illustrated by the apparition of a second degradation peak at higher temperatures.

After the degradation, systems immersed in CaCl_2_ presented higher residues than unreacted ones, probably due to the presence of calcium in the printed scaffold. Specifically, WBPUU0.5Ca30 and WBPUU0.5Ca60 showed a residue of about 2.5% and 3.5%, respectively, observing an increase of residue as the immersion time in CaCl_2_ increased, which can be correlated with the calcium content.

**Figure 9:**
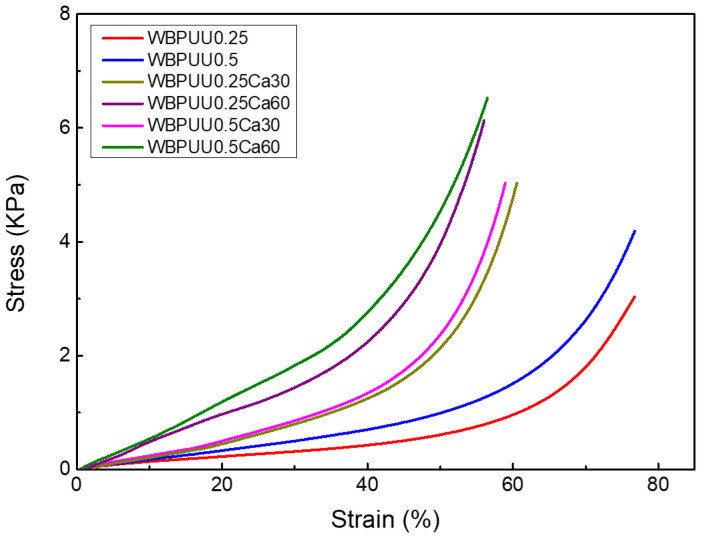
Stress–strain compression curves of WBPUU/CNC and WBPUU/CNC/CaCl_2_ scaffolds.

Compression tests were carried out on the scaffolds of the different systems that showed good shape fidelity to study the influence of calcium crosslinking and the addition of CNC on the mechanical properties. The measured values are summarized in Table 4, while a representative stress–strain curve for each system is shown in Figure 9. As can be observed in the figure, most systems presented typical compression behavior of porous materials, showing an initial linear elastic response followed by an extended plateau and a final region of increasing stress [65,66,67]. However, systems immersed in CaCl_2_ did not present the aforementioned plateau, presenting a continuous increase of the stress with the strain. This behavior, which is more typical of non-porous material, can be attributed to the compact shell formed through Ca^2+^ crosslinking. In systems not immersed in CaCl_2_, to the contrary, initial bending and buckling happened on the walls of the scaffold as the compression force was applied, but some cracking initiated at the yield point and the plateau is the result of progressive brittle crushing of the pores under the compressive loads. In the final region, densification of the porous structure pilots the behavior of the scaffold, becoming closer to that of a non-porous system.

Comparing the obtained compression values, it can be observed that the increase of the CNC content in the scaffolds resulted in an increase of both the Young’s modulus and stress. The presence of the CNC in the scaffold led to obtaining stiffer materials. Moreover, a decrease in the densification was observed as the CNC content increased. The reinforcement effect of the CNC can be corroborated with the comparison of the specific modulus and stress, without considering the effect of density. These results are also in accordance with those reported by Septevani et al., who observed an enhancement of the compression modulus and compressive strength with the addition of CNC to a polyurethane foam [68]. In the same context, Ugarte et al. explored the effect of the incorporation of CNCs into a polyurethane foam at an early stage of the synthesis [69], reporting an enhancement of the Young’s modulus with the addition of CNC.

Regarding the samples immersed on CaCl_2_, the previously observed rigid and non-porous shell resulted in a higher increase of the Young’s modulus. Indeed, an increase of 242% was observed comparing the Young’s moduli of WBPUU0.25 (19 ± 2 KPa) and WBPUU0.25Ca60 (65 ± 7 KPa). Additionally, as was previously mentioned, samples immersed in CaCl_2_ did not present the aforementioned plateau in stress–strain curves, with a continuous increase of the stress as the strain did. For Ca^2+^ crosslinked systems containing CNC, a synergic effect can be observed between both. On the one hand, due to the shell formed via crosslinking, the material acted as a non-foamed one due to the absence of porosity, and hence it is capable of better distributing stress, supporting higher stresses, and therefore, a higher modulus.

Regarding the densification strain of crosslinked scaffolds, lower values were obtained compared to not crosslinked ones, attributed to the lower cell size observed by SEM and the non-foamed part of the shelled systems. The obtained values of densification strain for crosslinked systems with CNC were lower compared with the ones observed in the literature for other polyurethane scaffold systems [70]. These results illustrate the obtaining of scaffolds with high rigidity, as a result of both the addition of CNC and the immersion in CaCl_2_.

**Figure 10:**
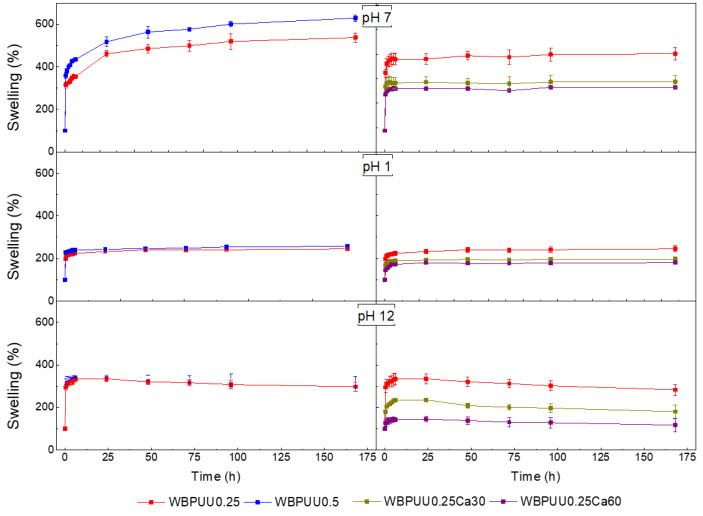
Water absorption capacity of the prepared WBPUU/CNC- and WBPUU/CNC/CaCl_2_-based scaffolds.

Figure 10 shows the water absorption capacity evolution of the prepared scaffolds at different pH levels. The results showed a variation of the WAC with the CNC content, the pH of the medium, and the immersion time in CaCl_2_. Additionally, the integrity of the scaffolds immersed in the different media was also studied by visual inspection to ensure their viability for the application at those conditions.

Concerning the CNC content, the water absorption capacity in all studied media increased as the CNC content did, showing a maximum WAC of 629% for the WBPUU0.5 system at pH = 7 after 1 week. WBPUU/CNC nanocomposites showed a greater water absorption capacity, because water molecules tend to diffuse where CNC nano-entities are located [71], and due to the hydrophilic character of CNC themselves [72,73,74]. In this context, an increase of 17% was observed at pH = 7 and 1 week from WBPUU0.25 to WBPUU0.5. Additionally, the 3D highly porous structure presented by samples containing CNC, as seen by SEM, and the subsequently high surface area, also contributed to the observed increase of the WAC. Regarding the samples immersed in CaCl_2_, the WAC decreased as the immersion time increased. The crosslinking through Ca^2+^ addition and the resulting rigid shell at the surface of the cylinder decreased the porosity, as was observed by SEM, which justifies the reduction of the WAC observed in all media. Indeed, the WBPUU0.25Ca60 presented a decrease of around 35% with respect to the WBPUU0.25 at pH = 7.

Regarding the effect of the pH, variations can also be observed as far as WAC is concerned. The scaffolds immersed in pH = 7 medium presented a higher WAC capacity compared with the other ones. For example, in the case of WBPUU0.25, the WCA was approximately 60% and 50% lower at pH = 1 and pH = 12, respectively. It should be noted that in basic medium, all samples showed a decrease in WAC from the second day of immersion, suggesting a disintegration of the scaffold in basic medium. This phenomenon, confirmed by visual examination, was not observed for the samples exposed to acid or neutral media. Similar behaviors have been reported in the literature for polyurethane-based systems, presenting good resistance to some acid media, but dissolving in basic ones [75,76,77].

## 4. Conclusions

In this work, shape-customized scaffolds based on WBPUU/CNC were successfully prepared with different CNC content by DIW 3D-printing and freeze-drying techniques. As a result, porous scaffolds with the desired shape were obtained. Additionally, some of the printed pieces were immersed in CaCl_2_ before freeze-drying, thus obtaining printing scaffolds with a rigid shell as a result of the chemical crosslinking between the Ca^2+^ and the carboxylate groups of the WBPUU particles. Scaffolds were tested by studying their mechanical properties, water absorption capacity, morphology, and thermal stability to analyze the effect of the crosslinking and CNC addition on the properties of the printed scaffolds.

The results showed that the addition of CNC to the prepared scaffolds provided a higher thermal stability as well as a higher compression modulus, resulting in systems that are more rigid. Moreover, the addition of CNC resulted in: (i) an increase of the shape fidelity of the inks, (ii) scaffolds that are able to better maintain the desired form, and (iii) a more homogeneous porous structure after the freeze-drying process, with a smaller pore size. Regarding the water absorption capacity of the WBPUU/CNC scaffolds, the aforementioned homogeneous porous structure with a higher specific surface resulted in an increase of the WAC as the CNC content increased.

The pieces immersed in CaCl_2_, on the contrary, showed a rigid shell surrounding the porous scaffold, which contributed to an increase of the compression modulus. The formed solid shell governs the mechanical properties. However, this external shell hampered the WAC, showing lower absorption values compared with the systems not crosslinked with CNC. Additionally, the obtained crosslinking showed that calcium ions also slightly increased the thermal stability of the scaffolds.

In conclusion, the combination of the DIW 3D-printing and the freeze-drying technology has been shown to be a successful way to obtain shape-customized scaffolds based on WBPUU. With the addition of CNC and the crosslinking produced by the immersion of the piece in CaCl_2_, the mechanical properties and swelling capacity of the scaffold can be tailored, depending on the needs of the material, thus expanding the potential of this type of material for applications requiring stiffer scaffolds with different water absorption capacities.

## Figures and Tables

**Table 1:** Designation, CNC content, and immersion time in CaCl_2_ of the prepared scaffolds.

System	CNC Content (%)	CaCl_2_ Immersion Time (min)
WBPUU	0	-
WBPUU0.25	0.25	-
WBPUU0.5	0.5	-
WBPUUCa30	0	30
WBPUU0.25Ca30	0.25	30
WBPUU0.5Ca30	0.5	30
WBPUUCa60	0	60
WBPUU0.25Ca60	0.25	60
WBPUU0.5Ca60	0.5	60

**Table 2:** Composition of the core and the shell of the WBPUU0.5Ca60 scaffold determined by EDS.

Analyzed Area	Composition		Spectrogram
	Element	(wt.%)	
Core	O	32.79	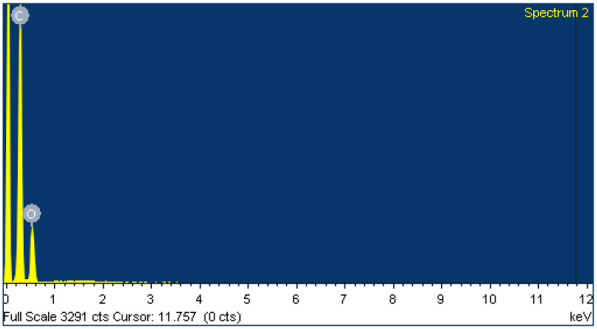
	C	73.19	
	Ca	-	
	Cl	-	
Shell	O	54.76	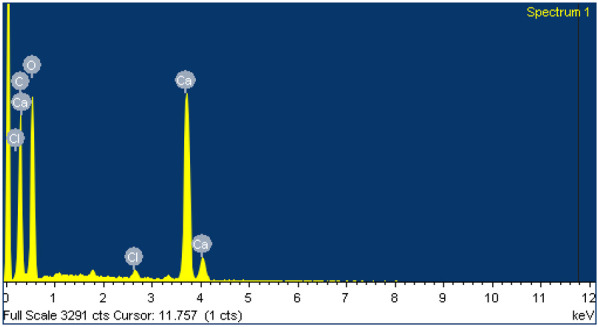
	C	29.68	
	Ca	5.98	
	Cl	0.52	

**Table 3:** Assignments of FTIR bands for WBPUU and WBPUU crosslinked with Ca^2+^.

Wavelength (cm^−1^)	Group	Type of Vibration	References
3348	N-H (Urethane and Urea)	Stretching	[49]
1735	C=O (Urethane)	Stretching	[50,51]
1640	C=O (Urea)	Stretching	[50,51]
1550	C–N (Urethane)	Stretching	[52,53]
	N-H (Urethane)	Symmetric bending	[54]
1460	C–N (Urea)	Stretching	[55,56]
	N–H (Urea)	Symmetric bending	
1250	C–N	Stretching	[57]
1150	CO–O	Asymmetric stretching	[58]
1024	CO–O	Symmetric stretching	[58]
1648	COO–Ca (asymmetric)	Asymmetric stretching	[59,60,61]
1420	COO–Ca (symmetric)	Symmetric stretching	[59,60,61]

**Table 4:** Density, compression properties, and specific compression properties of the WBPUU/CNC prepared scaffolds.

System	ρ (kg m^−3^)	E (KPa)	E/ρ (KPa/(kg m^−3^))	σ_40%_ (KPa)	σ_40%_/ρ (KPa/(kg m^−3^))	ε _densification_ (%)
WBPUU0.25	414 ± 3	19 ± 2	0.046 ± 0.005	418 ± 21	1.011 ± 0.045	65 ± 2
WBPUU0.25Ca30	484 ± 10	59 ± 1	0.121 ± 0.011	569 ± 17	1.175 ± 0.013	60 ± 0
WBPUU0.25Ca60	491 ± 40	65 ± 7	0.134 ± 0.015	1630 ± 31	3.319 ± 0.063	45 ± 1
WBPUU0.5	424 ± 4	30 ± 5	0.071 ± 0.012	516 ± 38	1.218 ± 0.066	64 ± 3
WBPUU0.5Ca30	525 ± 10	74 ± 5	0.142 ± 0.009	1001 ± 34	1.906 ± 0.055	52 ± 3
WBPUU0.5Ca60	537 ± 7	85 ± 1	0.157 ± 0.002	2013 ± 37	3.747 ± 0.071	48 ± 1

## Data Availability

The data presented in this study are available upon request from the corresponding authors.
